# CirclizePlus: using ggplot2 feature to write readable R code for circular visualization

**DOI:** 10.3389/fgene.2025.1535368

**Published:** 2025-03-27

**Authors:** Zheyu Zhang, Tianze Cao, Yuexia Huang, Yu Xia

**Affiliations:** School of Mathematics, Hangzhou Normal University, Hangzhou, China

**Keywords:** circlize, ggplot2, object-oriented, generic functions, functional programming

## Abstract

In the R programming language, the *de facto* standard framework for drawing rectangular coordinates is ggplot2. The most important feature of ggplot2 is that it is object-oriented and uses the plus sign to overlay various objects. In the field of circular visualization, circlize is a popular software, but it is based on procedural programming. Making it object-oriented can make the logic of the written code clearer and improve the reusability of the code. In this work, we introduce circlizePlus, which redesigns the concepts in circular visualization into several R S4 classes. It also defines a set of additional rules, based on which users can implement ggplot2-like drawing techniques. circlizePlus is a wrapper for circlize. It transforms the procedural programming style of circular visualization drawing into object-oriented programming. The additional rules it defines reduce the amount of coding and make the code more readable. The source codes can be found at https://github.com/tianzelab/circlizePlus, and the sample code can be found at https://tianzelab.github.io/circlizePlusBook/.

## 1 Introduction

**FIGURE 1:**
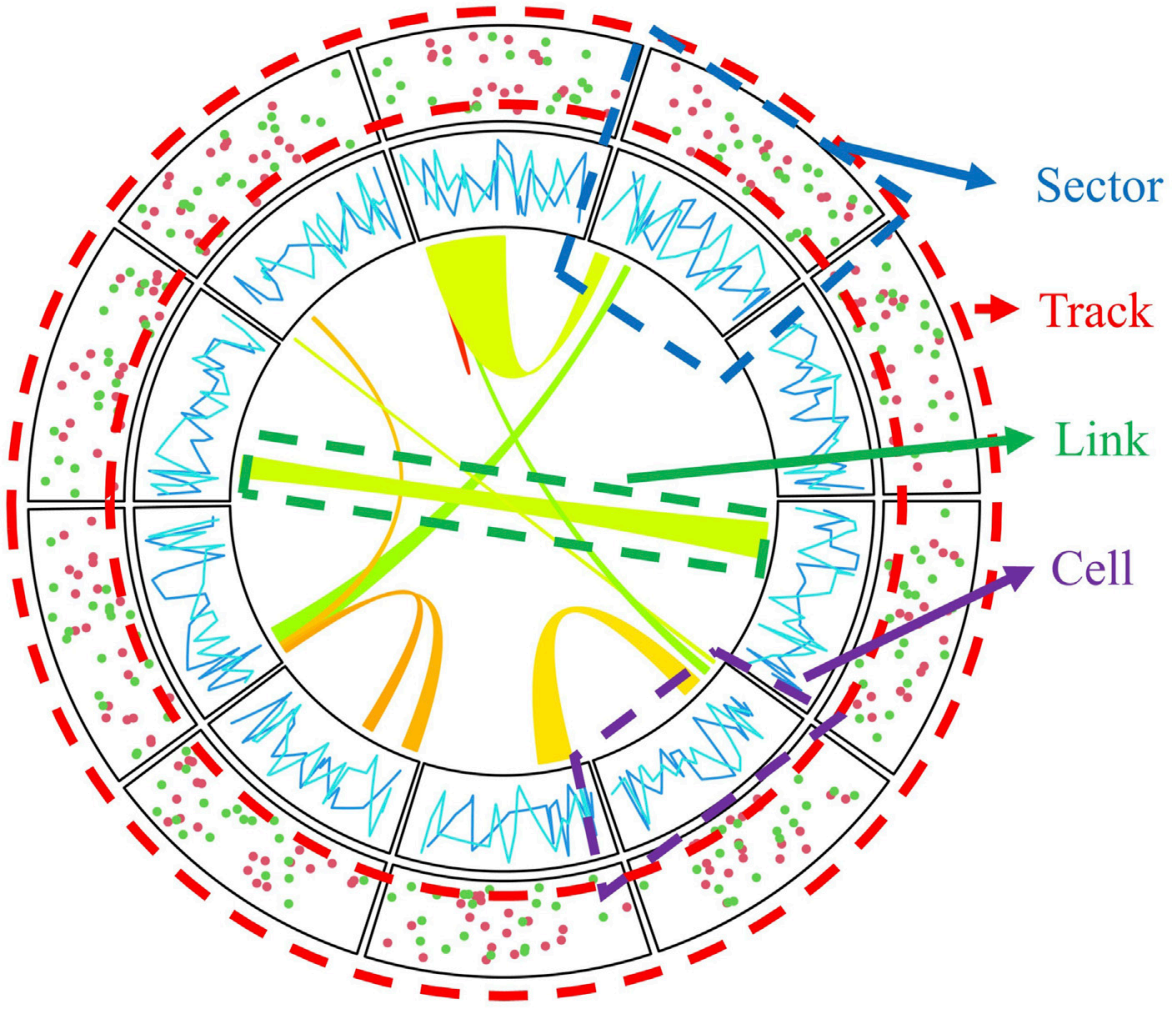
Schematic diagram of circle layout plotting.

Circular layout plots typically consist of sectors and tracks. The intersection of sectors and tracks is called a cell (Figure 1). This kind of layout can not only transparently represent the relationship between different data categories, but also rep-resent different observations from different dimensions within the same category. For instance, in genomics, different sectors may represent different chromosomes. In contrast, every single track layer can represent a different data category, such as gene density, or the variation standard, to name but a few.

Although numerous packages have been developed to implement circle layout plotting (Krzywinski et al., 2009; Darzentas, 2010; Zhang et al., 2013; Gu et al., 2014; An et al., 2015; Cheong et al., 2015; Cui et al., 2016; Diaz-Garcia et al., 2017; Drori et al., 2017; Yu et al., 2018; Marx and Coon, 2019; Cui et al., 2020; Rasche and Hiltemann, 2020; Cui et al., 2021; Ennis et al., 2023), circlize (Gu et al., 2014) has become the most mainstream tool since its release. It is an R package that offers various tools and functions to form these complex circle layout plots. Based on the statistical and graphical syntax of R, the package is especially easy for those who are good at R to use. By using circlize package, users can generate various types of charts, including scatter plots, histograms, line plots, heatmaps, etc.

By using circlize package, users can create many sorts of graphical elements easily, such as adding points, lines, texts, and axes. For instance, circos.points () function may be used for adding points, circos.line () for lines, and circos.text () for text labels. Moreover, the package is also accessible for stacking tracks and dividing sections, allowing every dataset to be displayed in the appropriate position.

In the domain of rectangular layout plotting, ggplot2 (Wickham, 2016) offers high-level flexibility and controllability by object-oriented programming and the overloading of the addition operator, which makes it much more popular than the basic R plotting API.

However, the classic way to use circlize is procedural programming, so it is necessary to provide support for object-oriented programming and addition operators like ggplot2. This will allow users to overlay different graphic elements in a more intuitive and modular way when creating complex circular layouts. Here, we’d like to introduce circlizePlus, which implements object-oriented programming and additive operations in circular layout plotting by wrapping circlize.

## 2 Materials and methods

### 2.1 Classes and addition rules in circlizePlus

The classes and object constructors defined in circlizePlus are prefixed with “cc,” to avoid naming conflicts with other packages and represent the abbreviation of circlize. circlizePlus defines 12 R S4 classes, 7 of which are primary classes, namely, ccPlot, ccPar, ccTrack, ccTrackGeom, ccLink, ccCell, and ccCellGeom. Based on these primary classes, circlizePlus constructs a set of addition operation rules as follows.

$$ccPlot\left(contain\;n\;ccPars\right)+ccPar=ccPlot\left(contain\;n+1\;ccPars\right),n\geq 0$$

$$ccPlot\left(contain\;n\;ccTracks\right)+ccTrack=ccPlot\left(contain\;n+1\;ccTracks\right),n\geq 0$$

$$ccPlot\left(contain\;n\;ccLinks\right)+ccLink=ccPlot\left(contain\;n+1\;ccLinks\right),n\geq 0$$

$$ccTrak\left(contain\;n\;ccTrakGeoms\right)+ccTrackGeom=ccTrack\left(contain\;n+1\;ccTrackGeoms\right),n\geq 0$$

$$ccTrack\left(contain\;n\;ccCells\right)+ccCell=ccTrack\left(contain\;n+1\;ccCells\right),n\geq 0$$

$$ccCell\left(contain\;n\;ccCellGeoms\right)+ccCellGeom=ccCell\left(contain\;n+1\;ccCellGeoms\right),n\geq 0$$

### 2.2 Data mapping from track to geometry

In ggplot2, the parameter data of the function plotting the geometry can be missing. In this case, the value of the parameter with the same name in the function ggplot () will be taken as the default value. Similar features are also implemented in circlizePlus. The coordinate parameters (such as x, y) of the function that draws geometric figures in the sector can be missing. circlizePlus will extract the data of the corresponding sector of the track it is added to and set it as the default value. It is worth noting that the coordinate parameter can be an anonymous function of the form “function (x, y) …”. This anonymous function will be called with the default parameter values above, and its re-turn value will be used as the actual value of the coordinate parameter.

### 2.3 Relationships between ccPar, ccTrack, ccLink and ccPlot

ccPlot is the core class of circlizePlus, whose objects are generated by the homonymous object constructor ccPlot (). The object of ccPlot acts as a container, holding the objects of ccPar, ccTrack, and ccLink. The objects of ccPar are generated by the homonymous object constructor ccPar(), defining the global parameter for plotting. Users may add one or more ccPar objects to a ccPlot object. circlizePlus aggregates multiple ccPar objects, thus determining the global plotting parameters. A single ccTrack object defines how to plot a track. There is also a subclass of ccTrack named ccGenomicTrack. Their object constructors and functionalities are listed in Supplementary Table S1. A single ccLink object stores data of the link connecting two sectors. There are also 2 subclasses of ccLink: ccHeatmapLink and ccGenomicLink. Their object constructors and functionalities are listed in Supplementary Table S2.

When plotting, users are supposed to call the generic show () function, in which ccPlot objects serve as a parameter. Once the show () function is called, circlizePlus will set global plotting parameters based on ccPar objects stored in ccPlot. Meanwhile, it plots tracks based on stored ccTrack objects or their subclasses, and plots lines based on ccLink objects or their subclasses.

### 2.4 Relationships between ccTrack, ccTrackGeom, ccCell and ccCellGeom

ccTrackGeom and ccCellGeom stored in ccTrack determine the geometrics plotted in the current track. Data stored in ccTrackGeom works on the entire track, while data stored in ccCellGeom works only on a single cell within the track. ccCellGeom objects cannot be add directly into ccTrack. It must first be added to a ccCell object before adding the ccCell object to ccTrack thereafter. ccCellGeom has a subclass named ccGenomicCellGeom. The object constructors and functionalities of ccTrackGeom and ccCellGeom are listed in Supplementary Table S3.

## 3 Results

### 3.1 Workflow of circlizePlus

**FIGURE 2:**
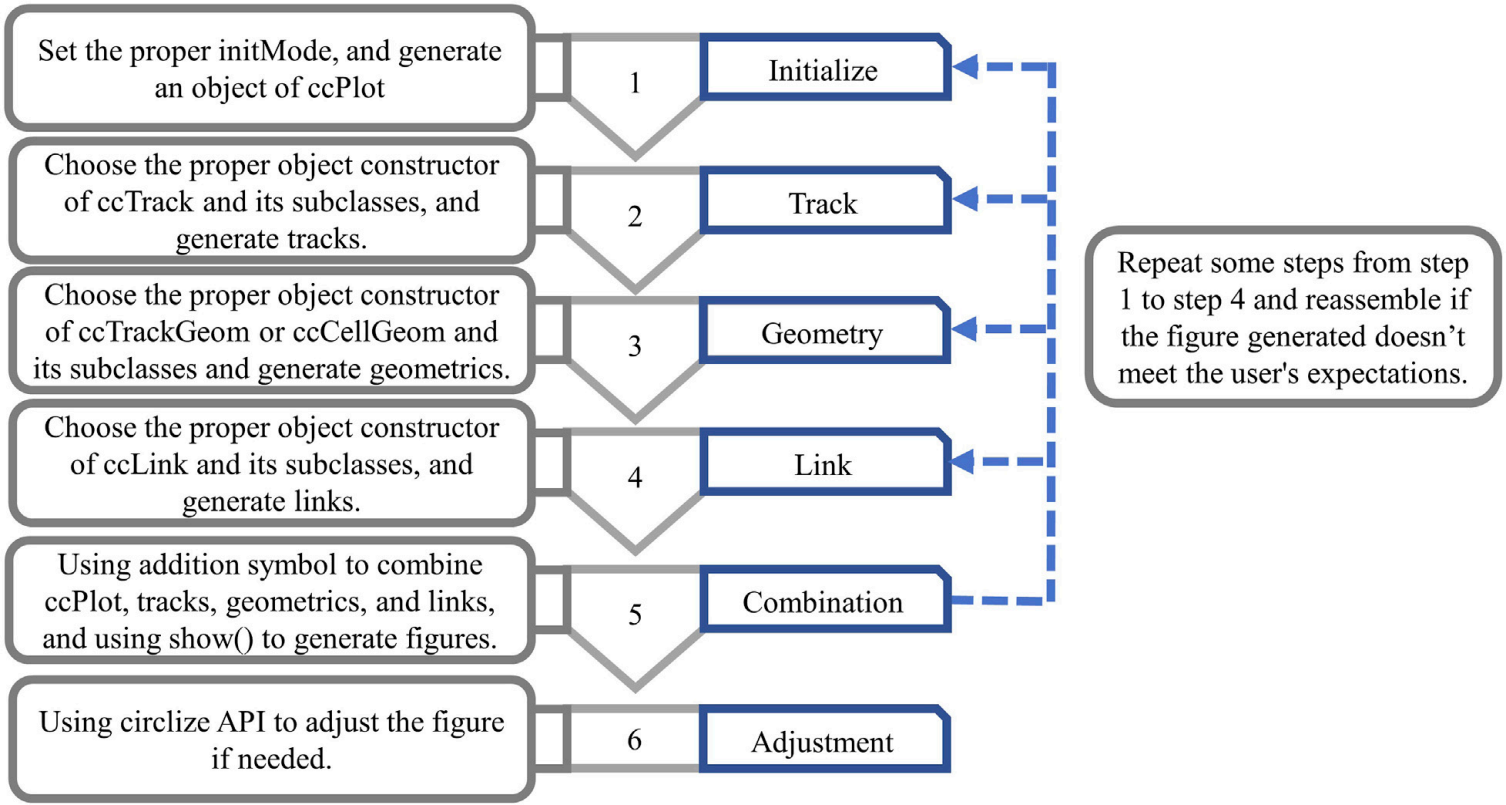
Workflow diagram of circlizePlus.

There are approximately 6 steps for users using circlizePlus to plot (Figure 2), with no strict order restriction for steps 1 to 4.

Step 1: When initiating plot programming, users must first call the ccPlot () function to generate a ccPlot object. Users also need to set a mandatory parameter named “initMode,” which can take one of the following 4 values: “initialize,” “heatmap.initialize,” “initializeWithIdeogram” or “genomicInitialize.” Different values of initMode correspond to different plotting scenes. The backend of circlizePlus will call the corresponding initialization functions from the circlize package based on the value of initMode. Therefore, ccPlot () function will have different applicable parameters.
Step 2: For general plotting, users are supposed to generate a ccTrack object. As for visualizing genomic data, users are supposed to create a ccGenomicTrack object.
Step 3: Users are supposed to select the appropriate object constructor function to generate geometric objects, according to the geometrics to be generated.
Step 4: Users are supposed to generate ccLink objects or one of its subclasses, in case users need to connect sectors to represent the relationships between each of them.
Step 5: Users assemble objects according to the addition rules of circlizePlus, and finally call the generic show () function, in which the ccPlot object is used as a parameter to generate a graph. Users are supposed to repeat some steps in steps 1 to 4 and reassemble if the figure generated does not meet expectations.
Step 6: circlizePlus is compatible with the circlize API. If the figure obtained by the above steps is not satisfactory, the user can also call the circlize API for further fine-tuning.

### 3.2 Example 1: plotted dot plot categorized by chromosome, and zoomed for partial chromosome categorization

**FIGURE 3:**
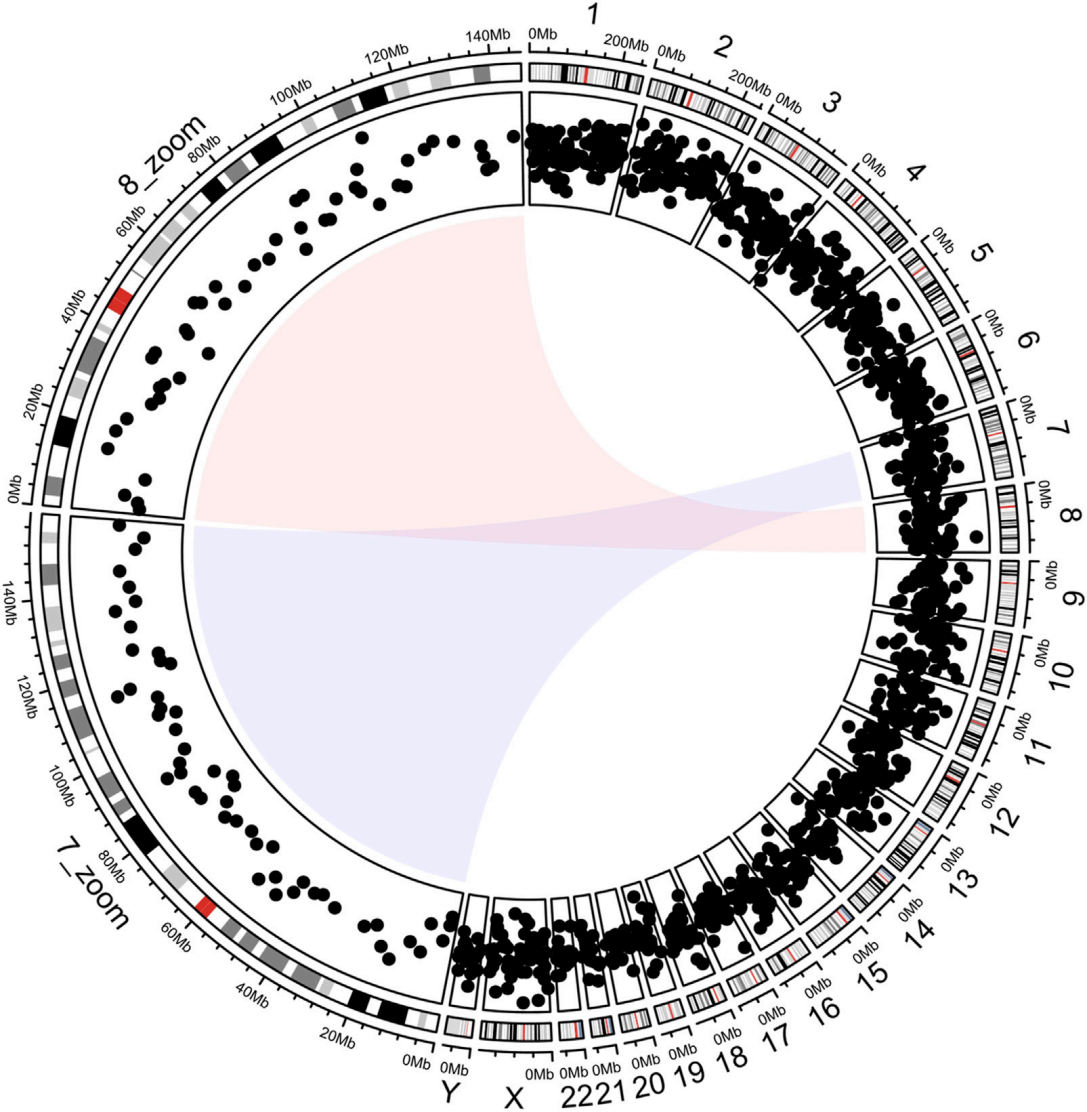
Chromosomes 7 and 8 are shown enlarged on the left half of the circle.

This section demonstrates the workflow of circlizePlus based on a small case (Gu et al., 2014). The code lines are segmented according to the workflow defined in the article (codes are shown in Supplementary File S1). It plots the chromosome bands in the outer circle and the corresponding scatter points in the inner circle (Figure 3). Magnified views of chromosomes 7 and 8 are shown on the left parts of the circles. The entire code process is like solving an addition problem, and the data flow of drawing can be clearly understood. Note that the data mapping technique is used in step 3. The coordinate parameter of the function ccGenomicPoints () is missing. It will take the coordinate parameter value of the corresponding sector from the variable track1. This technique ensures consistency of coordinate data while reducing code duplication.

## 4 Discussion

### 4.1 Why use object-oriented programming

circlizePlus is implemented using object-oriented programming. It is class- and object-centric, with an emphasis on data encapsulation and behavioral reuse. The resulting figure is ultimately determined by the property values of all objects used for drawing, making it easy to maintain and expand.

circlize, on the other hand, is implemented using traditional process-oriented programming, which has functions and processes at its core, and figures are the result of sequential execution of functions. It's suitable for running step by step, with each step calling a function and the corresponding changes will be made immediately on the figure. However, when the parameters that need to be adjusted are used by functions that have been called in some previous steps, the user often has to rerun all the previous code. For example, Figure 3 can be plotted using either circlize or circirclizePlus. When it comes to adjusting the width of the outermost track, circlizePlus needs to run less code, because circlize needs to rerun the code from scratch. In summary, circlizePlus offers advantages in terms of maintenance and scalability, while circlize is better suited for scenarios that require real-time feedback and single-step commissioning (codes are shown in Supplementary File S2).

### 4.2 Example 2: comparison of two pieces of code that use circlizePlus and circlize to implement the same requirements, respectively

**FIGURE 4:**
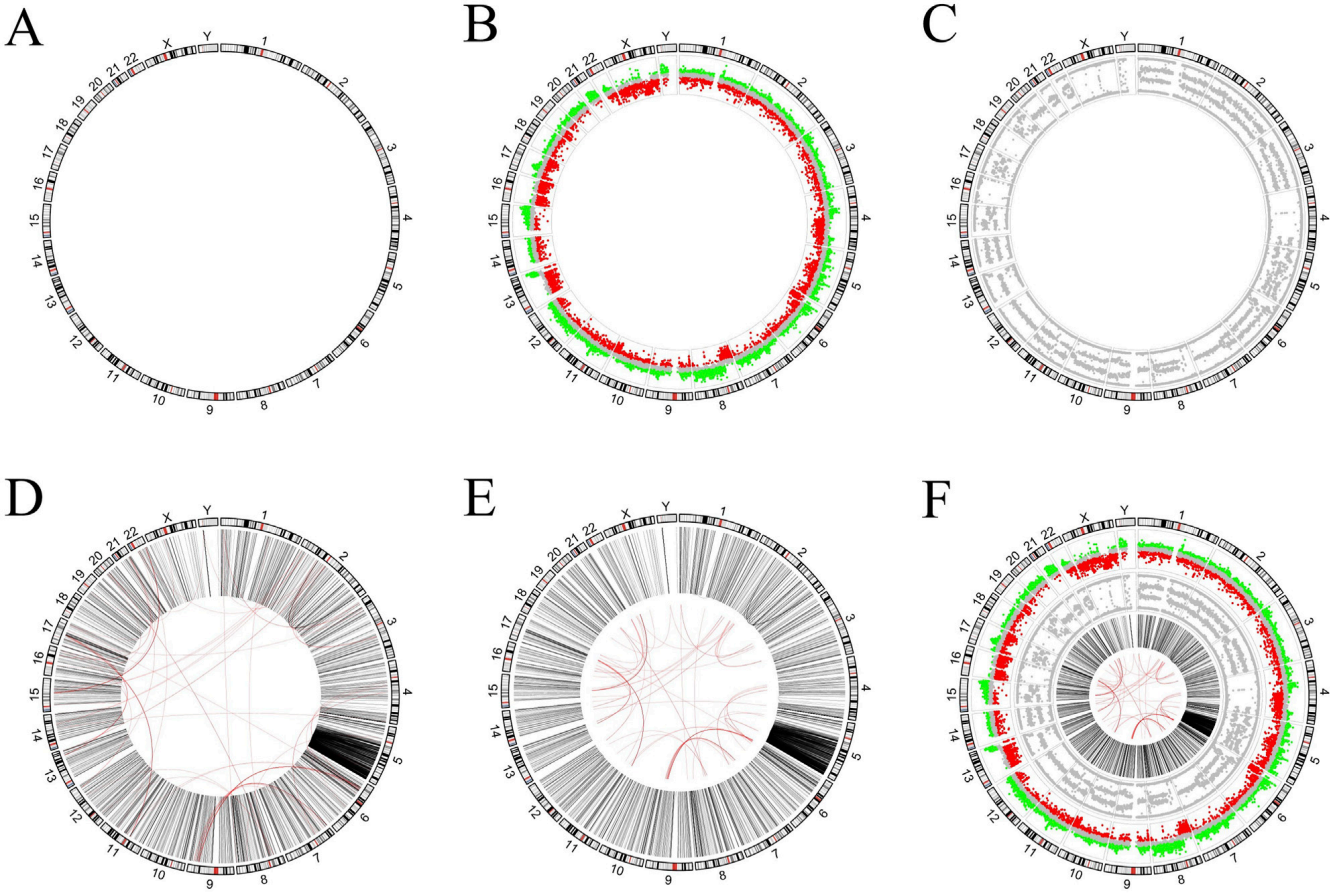
Figure generated in example 2. **(A)** Chromosome ideograms in humans. **(B)** The outer ring is the chromosome ideograms, and the inner ring is the dot plot, which is used to represent the number of copies of the gene on the corresponding chromosome. **(C)** The inner ring is also a dot plot that is used to represent the B-allele frequency of the corresponding chromosome. **(D)** The inner loop is a linked line diagram that is used to represent structural variations. **(E)** For aesthetic reasons, the red link lines in Figure D have been scaled. **(F)** The resulting diagram obtained by combining all of the above plots.

In this section, we try to draw pictures from real publications. The literature (Alves et al., 2013) uses a plot similar to Figure 4 to present the VCaP cancer cell line. The outermost ring corresponds to human chromosome ideograms (Figure 4A). It serves as an X-axis to indicate the position of the data of other rings on the chromosome. The inner ring in Figure 4B represents the number of gene copies, and the value of each dot is from the Affymetrix SNP arrays. Its Y-axis range is −1 to 1, where dots from 0.15 to 1 are marked in red, dots from −0.15 to 0.15 are marked in grey, and dots from −1 to 0.15 are marked in green. The inner ring in Figure 4C represents the B-allele frequency (ratio), which ranges from 0 to 1 on the Y-axis. The links plot is used to reflect structural variations (SVs) (Figure 4D). The intra- and inter-chromosomal SVs are on the inner rings and depicted in black and red lines, respectively. The red link lines are obscured by the black link lines because there are quite a few intra-chromosomal SVs. The red link lines have been reduced in scale (Figure 4E) for aesthetic reasons. Combining all of the above plots together gives the picture in the literature (Figure 4F).

The process of drawing Figure 4 is to draw the graphs of the various categories first, and then put them together. Quite a few researchers use this process to create circos diagrams. Both circlize and circlizePlus implement such a process (codes are shown in Supplementary File S3). According to code statistics, circlizePlus uses less code than circlize in such a process. Code statistics show that circlizePlus uses less code than circlize in such a process (Table 1).

**TABLE 1:** Statistics on the amount of code in Example2.

	Figure 4A	Figure 4B	Figure 4C	Figure 4D	Figure 4E	Figure 4F	Total
The number of lines of code implemented with circlize	3	10	6	6	6	16	48
The number of lines of code implemented with circlizePlus	2	8	4	4	4	4	28
The number of characters in the code implemented with circlize (excluding spaces)	118	407	303	277	291	765	2177
The number of characters in the code implemented with circlizePlus (excluding spaces)	100	329	291	175	189	230	1359

## Data Availability

Publicly available datasets were analyzed in this study. This data can be found here: https://github.com/tianzelab/circlizePlus and https://tianzelab.github.io/circlizePlusBook/.
