# Supplementary material for: CirclizePlus: using ggplot2 feature to write readable R code for circular visualization
Source: Front Genet. 2025 Mar 27;16:1535368. doi: 10.3389/fgene.2025.1535368 (PMC11983637; doi:10.3389/fgene.2025.1535368)
Supplement: Supplementary file 1 [file Table1.docx]

Table S1 Constructors of ccTrack and its subclasses

| Constructor name | Class returned | Description |
| --- | --- | --- |
| ccTrack() | ccTrack | Define a generic track |
| ccTrackHist() | ccTrack | Define a track of histograms |
| ccGenomicTrack() | ccGenomicTrack | Define a track for genomic data visualization |
| ccGenomicIdeogram() | ccGenomicTrack | Define an ideograms track for genomic graph |
| ccGenomicHeatmap() | ccGenomicTrack | Define a heatmap track for genomic graph |
| ccGenomicLabels() | ccGenomicTrack | Add labels to specific genomic track |
| ccGenomicRainfall() | ccGenomicTrack | Create a rainfall plot |
| ccGenomicDensity() | ccGenomicTrack | Create a track of density plot |
