# Supplementary material for: CirclizePlus: using ggplot2 feature to write readable R code for circular visualization
Source: Front Genet. 2025 Mar 27;16:1535368. doi: 10.3389/fgene.2025.1535368 (PMC11983637; doi:10.3389/fgene.2025.1535368)
Supplement: Supplementary file 2 [file Presentation1.pdf]

Code used in Example 1

Step 1

```
library(circlizePlus)
cytoband = read.cytoband()
df = cytoband$df
chromosome = cytoband$chromosome
chr.len = cytoband$chr.len
df_zoom = df[df[[1]] %in% c("chr7", "chr8"), ]
df_zoom[[1]] = paste0(df_zoom[[1]], "_zoom")
df = rbind(df, df_zoom)
bed = generateRandomBed(nr = 1000)
bed_zoom = bed[bed[[1]] %in% c("chr7", "chr8"), ]
bed_zoom[[1]] = paste0(bed_zoom[[1]], "_zoom")
bed = rbind(bed, bed_zoom)

start90 = ccPar(start.degree = 90)
cc = ccPlot(initMode = "initializeWithIdeogram", cytoband = df, sort.chr = FALSE,
sector.width = c(chr.len/sum(chr.len), 0.5, 0.5))
```

Step 2

```
trak1 = ccGenomicTrack(data = bed)
```

Step 3

```
all_cell = ccCells(sector.indexes = unique(df[[1]])) + ccGenomicPoints(pch = 16,
cex = 0.8)
trak1 = trak1 + all_cell
```

Step 4

```
chr7_x_start = min(df[which(df$V1 == 'chr7'),2])
ch7_x_end = max(df[which(df$V1 == 'chr7'),3])
link_ch7_to_zomm = ccLink("chr7", c(chr7_x_start, ch7_x_end), "chr7_zoom",
c(chr7_x_start, ch7_x_end), col = "#0000FF10", border = NA)

chr8_x_start = min(df[which(df$V1 == 'chr8'),2])
ch8_x_end = max(df[which(df$V1 == 'chr8'),3])
link_ch8_to_zomm = ccLink("chr8", c(chr8_x_start, ch8_x_end), "chr8_zoom",
c(chr8_x_start, ch8_x_end), col = "#FF000010", border = NA)
```

Step 5

```
show(cc + start90 + trak1 + link_ch7_to_zomm + link_ch8_to_zomm)
```
