# Supplementary material for: CirclizePlus: using ggplot2 feature to write readable R code for circular visualization
Source: Front Genet. 2025 Mar 27;16:1535368. doi: 10.3389/fgene.2025.1535368 (PMC11983637; doi:10.3389/fgene.2025.1535368)
Supplement: Supplementary file 3 [file Table2.docx]

Table S2 Constructors of ccLink and its subclasses

| Constructor name | Class returned | Description |
| --- | --- | --- |
| ccLink() | ccLink | Add a link |
| ccHeatmapLink() | ccHeatmapLink | Add a link between rows in a heatmap |
| ccGenomicLink() | ccGenomicLink | Add links between two sets of genomic positions |
