# Supplementary material for: CirclizePlus: using ggplot2 feature to write readable R code for circular visualization
Source: Front Genet. 2025 Mar 27;16:1535368. doi: 10.3389/fgene.2025.1535368 (PMC11983637; doi:10.3389/fgene.2025.1535368)
Supplement: Supplementary file 4 [file Table3.docx]

Table S3 Constructors of ccTrackGeom, ccCell, ccCellGeom and their subclasses

| Constructor name | Class returned | Description |
| --- | --- | --- |
| ccCell() | ccCell | Generate a cell container that belongs to a particular sector |
| ccText() | ccCellGeom | Draw text in a cell |
| ccPoints() | ccCellGeom | Draw a point in a region |
| ccLines() | ccCellGeom | Draw lines in a region |
| ccSegments() | ccCellGeom | Draw segments connecting points in a region |
| ccRect() | ccCellGeom | Draw rectangle in a region |
| ccPolygon() | ccCellGeom | Draw polygon |
| ccXaxis() | ccCellGeom | Draw x-axis |
| ccYaxis() | ccCellGeom | Draw y-axis |
| ccBarplot() | ccCellGeom | Draw bar-plots |
| ccViolin() | ccCellGeom | Draw violin plots |
| ccArrow() | ccCellGeom | Draw an arrow |
| ccRaster() | ccCellGeom | Add raster image |
| ccDendrogram() | ccCellGeom | Draw dendrogram plots in a track |
| ccGenomicPoints() | ccGenomicCellGeom | Add points for genomic data visualization |
| ccGenomicLines() | ccGenomicCellGeom | Add lines for genomic data visualization |
| ccGenomicRect() | ccGenomicCellGeom | Draw rectangle for genomic data visualization |
| ccGenomicText() | ccGenomicCellGeom | Add text for genomic data visualization |
| ccGenomicAxis() | ccGenomicCellGeom | Add genomic axes |
| ccTrackLines() | ccTrackGeom | Add lines on all sections of a single track. |
| ccTrackPoints() | ccTrackGeom | Add points on all sections of a single track. |
| ccTrackText() | ccTrackGeom | Add texts on all sections of a single track. |
