# Supplementary material for: CirclizePlus: using ggplot2 feature to write readable R code for circular visualization
Source: Front Genet. 2025 Mar 27;16:1535368. doi: 10.3389/fgene.2025.1535368 (PMC11983637; doi:10.3389/fgene.2025.1535368)
Supplement: Supplementary file 5 [file Presentation2.pdf]

# Code used in the section "Why use object-oriented programming"

## 1 Based on circlize, draw Figure 3, adjust the height of the inner track, and redraw

### 1.1 Draw Figure 3 based on circlize

```
library(circlize)
cytoband = read.cytoband()
df = cytoband$df
chromosome = cytoband$chromosome
chr.len = cytoband$chr.len

df_zoom = df[df[[1]] %in% c("chr7", "chr8"), ]
df_zoom[[1]] = paste0(df_zoom[[1]], "_zoom")
df = rbind(df, df_zoom)

bed = generateRandomBed(nr = 1000)
bed_zoom = bed[bed[[1]] %in% c("chr7", "chr8"), ]
bed_zoom[[1]] = paste0(bed_zoom[[1]], "_zoom")
bed = rbind(bed, bed_zoom)

circos.par(start.degree = 90)
circos.initializeWithIdeogram(df, sort.chr = FALSE, sector.width =
c(chr.len/sum(chr.len), 0.5, 0.5))
circos.genomicTrackPlotRegion(bed, panel.fun = function(region, value, ...) {
    circos.genomicPoints(region, value, pch = 16, cex = 0.8)
})

circos.link("chr7", get.cell.meta.data("cell.xlim", sector.index = "chr7"),
            "chr7_zoom", get.cell.meta.data("cell.xlim", sector.index =
"chr7_zoom"),
            col = "#0000FF10", border = NA)
circos.link("chr8", get.cell.meta.data("cell.xlim", sector.index = "chr8"),
            "chr8_zoom", get.cell.meta.data("cell.xlim", sector.index =
"chr8_zoom"),
            col = "#FF000010", border = NA)
circos.clear()
```

### 1.2 The code used to adjust the height of the inner track in the figure

```
circos.par(start.degree = 90)
circos.initializeWithIdeogram(df, sort.chr = FALSE, sector.width =
c(chr.len/sum(chr.len), 0.5, 0.5))
circos.genomicTrackPlotRegion(bed, track.height=0.4, panel.fun = function(region,
value, ...) {
    circos.genomicPoints(region, value, pch = 16, cex = 0.8)
```

```
})

circos.link("chr7", get.cell.meta.data("cell.xlim", sector.index = "chr7"),
            "chr7_zoom", get.cell.meta.data("cell.xlim", sector.index =
"chr7_zoom"),
            col = "#0000FF10", border = NA)
circos.link("chr8", get.cell.meta.data("cell.xlim", sector.index = "chr8"),
            "chr8_zoom", get.cell.meta.data("cell.xlim", sector.index =
"chr8_zoom"),
            col = "#FF000010", border = NA)
circos.clear()
```

# 2 Based on circlizePlus, draw Figure 3, adjust the height of the inner track, and redraw

## 2.1 Draw Figure 3 based on circlizePlus

```
library(circlizePlus)
cytoband = read.cytoband()
df = cytoband$df
chromosome = cytoband$chromosome
chr.len = cytoband$chr.len
df_zoom = df[df[[1]] %in% c("chr7", "chr8"), ]
df_zoom[[1]] = paste0(df_zoom[[1]], "_zoom")
df = rbind(df, df_zoom)
bed = generateRandomBed(nr = 1000)
bed_zoom = bed[bed[[1]] %in% c("chr7", "chr8"), ]
bed_zoom[[1]] = paste0(bed_zoom[[1]], "_zoom")
bed = rbind(bed, bed_zoom)
start90 = ccPar(start.degree = 90)
cc = ccPlot(initMode = "initializeWithIdeogram", cytoband = df, sort.chr =
FALSE,sector.width = c(chr.len/sum(chr.len), 0.5, 0.5))
trak1 = ccGenomicTrack(data = bed)
all_cell = ccCells(sector.indexes = unique(df[[1]])) + ccGenomicPoints(pch =
16,cex = 0.8)
trak1 = trak1 + all_cell
chr7_x_start = min(df[which(df$V1 == 'chr7'),2])
ch7_x_end = max(df[which(df$V1 == 'chr7'),3])
link_ch7_to_zomm = ccLink("chr7", c(chr7_x_start, ch7_x_end), "chr7_zoom",
c(chr7_x_start, ch7_x_end), col = "#0000FF10", border = NA)
chr8_x_start = min(df[which(df$V1 == 'chr8'),2])
ch8_x_end = max(df[which(df$V1 == 'chr8'),3])
link_ch8_to_zomm = ccLink("chr8", c(chr8_x_start, ch8_x_end),
"chr8_zoom",c(chr8_x_start, ch8_x_end), col = "#FF000010", border = NA)
show(cc + start90 + trak1 + link_ch7_to_zomm + link_ch8_to_zomm)
```

## 2.2 The code used to adjust the height of the inner track in the figure

```
trak1 = ccGenomicTrack(data = bed, track.height=0.4)
```

```
show(cc + start90 + (trak1+all_cell) + link_ch7_to_zomm + link_ch8_to_zomm)
```
