# Supplementary material for: CirclizePlus: using ggplot2 feature to write readable R code for circular visualization
Source: Front Genet. 2025 Mar 27;16:1535368. doi: 10.3389/fgene.2025.1535368 (PMC11983637; doi:10.3389/fgene.2025.1535368)
Supplement: Supplementary file 6 [file Presentation3.pdf]

Code used in Example 2

circlizePlus

```
params = ccPar(start.degree=90)
data("example2")
#Fig. 4A
cc = ccPlot(initMode="initializeWithIdeogram", species ="hg18", plotType = c("ideogram",  "labels"))
cc + params
#Fig. 4B
copy_number_track=ccGenomicTrack(data=copy_number, ylim=c(-1,1), numeric.column = 4, bg.lwd=0.1, panel.fun =
function(region, value, ...) {
    colors=value
    colors$col="gray"
    colors[colors$Value < -0.15,]$col="red"
    colors[colors$Value > 0.15,]$col="green"
    circos.genomicPoints(region, value, pch=20,cex=0.1, col = colors$col,...)
})
cc + params + copy_number_track
#Fig. 4C
allele_frequency_track=ccGenomicTrack(data=allele_frequency, ylim=c(0,1), numeric.column = 4, bg.lwd=0.1)
all_cell = ccCells(sector.indexes = unique(allele_frequency[[1]])) + ccGenomicPoints(pch=20,cex=0.1,col="gray")
allele_frequency_track=allele_frequency_track+all_cell
cc + params + allele_frequency_track
#Fig. 4D
junctions$col="black"
junctions[junctions$LeftChr!=junctions$RightChr,]$col="red"
links = ccGenomicLink(region1=r1, region2=r2,col=junctions$col,h.ratio=0.6, lwd=0.1)
cc + params + links
#Fig. 4E
junctions$rou=0.85
junctions[junctions$LeftChr!=junctions$RightChr,]$rou=0.45
links = ccGenomicLink(region1=r1, region2=r2,col=junctions$col,h.ratio=0.6, lwd=0.1, rou=junctions$rou)
cc + params + links
```

```
#Fig. 4F
junctions$rou=0.42
junctions[junctions$LeftChr!=junctions$RightChr,]$rou=0.22
links = ccGenomicLink(region1=r1, region2=r2,col=junctions$col,h.ratio=0.6, lwd=0.1, rou=junctions$rou)
cc + params +copy_number_track + allele_frequency_track + links
```

circlize

```
data("example2")
#Fig. 4A
circos.clear()
circos.par(start.degree=90)
circos.initializeWithIdeogram(species ="hg18", plotType = c("ideogram",  "labels"))
#Fig. 4B
circos.clear()
circos.par(start.degree=90)
circos.initializeWithIdeogram(species ="hg18", plotType = c("ideogram",  "labels"))
circos.genomicTrack(data=copy_number, ylim=c(-1,1), numeric.column = 4, bg.lwd=0.1, panel.fun = function(region, value, ...)
{
    colors=value
    colors$col="gray"
    colors[colors$Value < -0.15,]$col="red"
    colors[colors$Value > 0.15,]$col="green"
    circos.genomicPoints(region, value, pch=20,cex=0.1, col = colors$col,...)
})
#Fig. 4C
circos.clear()
circos.par(start.degree=90)
circos.initializeWithIdeogram(species ="hg18", plotType = c("ideogram",  "labels"))
circos.genomicTrack(data=allele_frequency, ylim=c(0,1), numeric.column = 4, bg.lwd=0.1, panel.fun = function(region, value,
...){
    circos.genomicPoints(region, value, pch=20,cex=0.1,col="gray",...)
})
#Fig. 4D
circos.clear()
```

```
circos.par(start.degree=90)
circos.initializeWithIdeogram(species ="hg18", plotType = c("ideogram",  "labels"))
junctions$col="black"
junctions[junctions$LeftChr!=junctions$RightChr,]$col="red"
circos.genomicLink(region1=r1, region2=r2,col=junctions$col,h.ratio=0.6, lwd=0.1)
#Fig. 4E
circos.clear()
circos.par(start.degree=90)
circos.initializeWithIdeogram(species ="hg18", plotType = c("ideogram",  "labels"))
junctions$rou=0.85
junctions[junctions$LeftChr!=junctions$RightChr,]$rou=0.45
circos.genomicLink(region1=r1, region2=r2,col=junctions$col,h.ratio=0.6, lwd=0.1, rou=junctions$rou)
#Fig. 4F
circos.clear()
circos.par(start.degree=90)
circos.initializeWithIdeogram(species ="hg18", plotType = c("ideogram",  "labels"))
circos.genomicTrack(data=copy_number, ylim=c(-1,1), numeric.column = 4, bg.lwd=0.1, panel.fun = function(region, value, ...)
{
    colors=value
    colors$col="gray"
    colors[colors$Value < -0.15,]$col="red"
    colors[colors$Value > 0.15,]$col="green"
    circos.genomicPoints(region, value, pch=20,cex=0.1, col = colors$col,...)
})
circos.genomicTrack(data=allele_frequency, ylim=c(0,1), numeric.column = 4, bg.lwd=0.1, panel.fun = function(region, value,
...){
    circos.genomicPoints(region, value, pch=20,cex=0.1,col="gray",...)
})
junctions$rou=0.42
junctions[junctions$LeftChr!=junctions$RightChr,]$rou=0.22
circos.genomicLink(region1=r1, region2=r2,col=junctions$col,h.ratio=0.6, lwd=0.1, rou=junctions$rou)
```
